# Extrapulmonary Sarcoidosis With Multi-Organ Involvement Presenting Primarily as Hypercalcemia

**DOI:** 10.7759/cureus.33562

**Published:** 2023-01-09

**Authors:** Inna Mikaella P Sta. Maria, Julia Tullio, Vasudevan Mahalingam, Jamal Abukhaled, Vamshi Garlapaty

**Affiliations:** 1 Department of Internal Medicine, Beaumont Health, Royal Oak, USA; 2 Department of Internal Medicine - Pediatrics, Beaumont Health, Royal Oak, USA; 3 Department of Pathology, Beaumont Health, Royal Oak, USA; 4 Department of Nephrology, Beaumont Health, Royal Oak, USA

**Keywords:** cutaneous sarcoidosis, hypercalcemia, sarcoidosis, extra pulmonary manifestations of sarcoidosis, multiorgan sarcoidosis

## Abstract

Sarcoidosis is a highly variable disease. The majority of cases affect the lungs, but they can involve other organs as well. Extrapulmonary sarcoidosis is rare, and it can present in many ways. Therefore, the diagnosis can be challenging. We hereby present a case of a patient presenting with hypercalcemia and diagnosed with extrapulmonary sarcoidosis with multi-organ involvement. This article was previously presented as a meeting abstract at the MI-ACP 2022 Annual Scientific Meeting on October 14, 2022.

## Introduction

Sarcoidosis is a highly variable, granulomatous disease. It most commonly affects females below 40 years of age [1]. Over 90% of the time, there is pulmonary involvement [2]. However, it is well documented that there are a variety of ways that sarcoidosis can present with extrapulmonary involvement. Mediastinal and hilar lymphadenopathy followed by cutaneous involvement is the most common extrapulmonary presentation [1]. African Americans are disproportionately affected by extrapulmonary manifestations [3].

Sarcoidosis is a diagnosis of exclusion. The major diagnostic criteria are as follows: compatible clinical presentation, finding non-necrotizing granulomatous inflammation in one or more tissue samples, and the exclusion of alternative causes of granulomatous disease [4]. Other causes of granulomatous disease that must be ruled out include lymphoproliferative disorders such as Hodgkin’s lymphoma and infectious diseases including leishmaniasis, toxoplasmosis, and tuberculosis [5].

Cutaneous sarcoidosis is described as specific or non-specific. Erythema nodosum is recognized as the most common non-specific skin manifestation of sarcoidosis. It can present as erythematous macules, papules, plaques, or subcutaneous nodules. It may also occur in scar tissue, at traumatized sites, and around embedded foreign bodies, such as tattoos. Reactions to tattoos may form in response to one or more colors within a tattoo and can develop even years after the placement of the tattoo [5-6]. These changes may even be seen in imaging, which is often used to evaluate the involvement of sarcoidosis. They appear as soft-tissue nodules or masses in the subcutaneous planes with low signal intensity on T1-weighted imaging and high signal intensity on T2-weighted imaging. Cutaneous findings should be taken as an opportunity to evaluate for systemic disease [7].

Sarcoidosis can also present with renal involvement, presenting as granulomatous interstitial nephritis and hypercalcemia [6,8]. Other extrapulmonary findings include ocular manifestations, most commonly uveitis. In addition, in the workup of lymphoproliferative disorders, granulomas can be seen on bone marrow biopsy, which is uncommon but documented in case reports [9]. Parotid gland enlargement can be seen on imaging and is usually bilateral. It is present in 6% of cases [10]. Other rare manifestations include splenomegaly, muscular involvement, and hepatic involvement.

The mainstay of treatment for all manifestations of sarcoidosis continues to be corticosteroids. In the setting of cutaneous sarcoidosis, topical corticosteroids can be used as well. Of note, methotrexate and antimalarials such as chloroquine and hydroxychloroquine have been shown to be good treatment options for cutaneous sarcoidosis and hypercalcemia [6].

## Case presentation

A 28-year-old African American woman presented to the emergency department with a four-month history of increased thirst, fatigue, intermittent headaches, myalgias, nausea, and a 20-lb unintentional weight loss. She has never been diagnosed with medical conditions requiring chronic medication use. She reported uveitis of the right eye in the summer of 2021, which was treated with topical steroids and subsequently improved. She was never prescribed a thiazide diuretic. She does not take antacids or herbal supplements. She does not have a significant smoking history. She works as an assembly line worker for a car company. She had similar symptoms two weeks prior to this admission and was found to have a high calcium level (16.3 mg/dL; reference range: 8.5-10.5 mg/dL) and a high ionized calcium level (7.36 mg/dL; reference range: 4.48-5.28 mg/dL). She was admitted, and investigations revealed a normal TSH (1.08 uIU/mL; reference range: 0.40-4.50 µIU/mL), low vitamin D, 25OH (9 ng/mL; reference range: 30-100 ng/mL), low vitamin A (20.1 mcg/dL; reference range: 32.5-78.0 mcg/dL), a serum protein electrophoresis with decreased total albumin and protein, and a high 24-hour urine calcium test (416 mg/24 hr; reference range: 100-300 mg/24 hr). AST was high (41 U/L; reference range: <35 U/L), while ALP and ALT were within normal limits. She was found to have an acute kidney injury secondary to pre-renal disease that improved with IV fluids. Calcium levels decreased, and she was discharged home with a plan to follow up outpatient for further workup of hypercalcemia. However, the same symptoms recurred before appointments could be arranged, along with additional symptoms of intermittent blurring of vision, dry eyes, and dry mouth.

On admission, she was found to have tachycardia and low blood pressure. A physical exam revealed no palpable cervical, supraclavicular, or axillary lymphadenopathy. The lungs were clear to auscultation bilaterally. There was a note of dense breast tissue on the upper outer quadrant of her left breast. An abdominal exam revealed a non-tender and non-distended abdomen with normoactive bowel sounds. She had no disturbance to consciousness and had the intact sensation of light touch and normal motor strength on all her extremities. Her tendon reflexes were normal, and there was no noted pathologic reflex.

**Figure 1:**
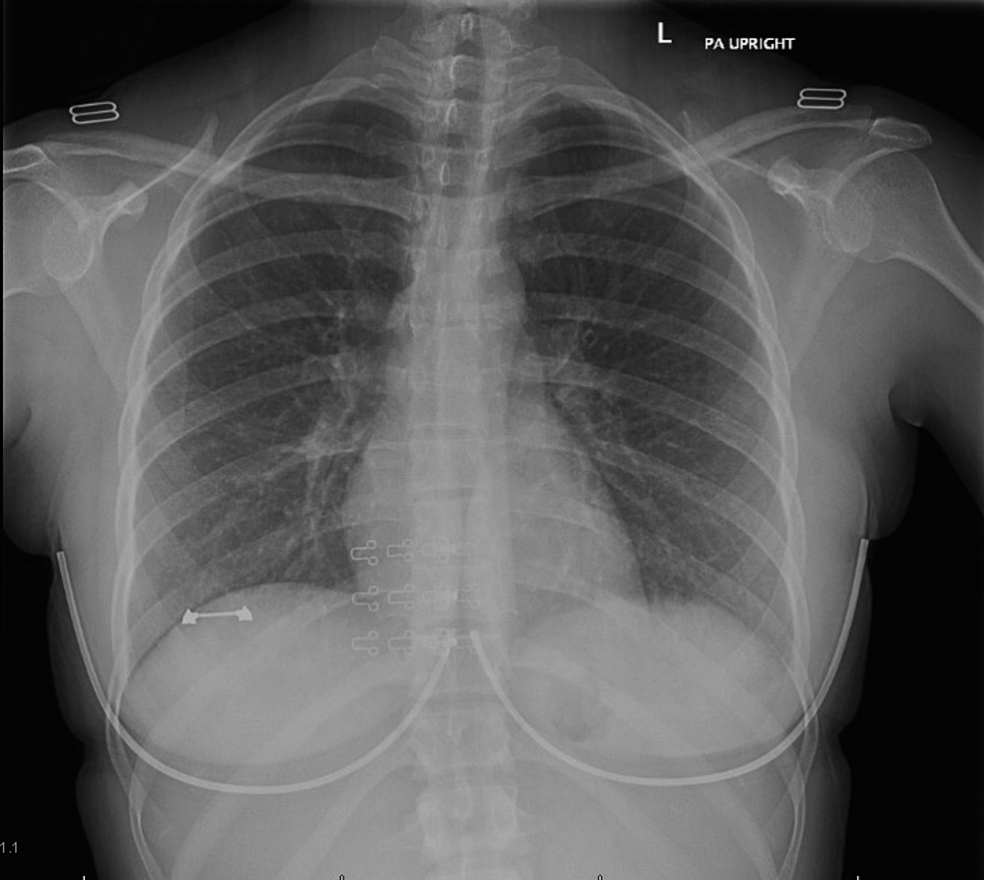
CXR demonstrating clear lungs and pleural spaces. No radiographic evidence of acute pulmonary disease.

Her laboratory testing revealed high levels of calcium (13.7 mg/dL; reference range: 8.5-10.5 mg/dL) but normal Parathyroid hormone (PTH; 8 pg/mL; reference range: 8-72 pg/mL) and PTH-related peptide (PTHrp) levels (0.9 pmol/L; reference range: ≤4.2 pmol/L). Given the initial laboratory results, there was a suspicion of sarcoidosis, and so an angiotensin-converting enzyme (ACE) level and an IL-2 (interleukin-2 receptor) level were checked, both of which were found to be high. The ACE level was >120 U/L (reference range: 7-60 U/L) and the IL-2 level was 3654.5 pg/mL (reference range: 175.3-858.2 pg/mL). Further work-up revealed an elevated creatinine (1.43 mg/dL; reference range: 0.50-1.10 mg/dL), high vitamin D-1,25 OH (172 pg/mL; 19.9-79.3 pg/mL), negative Sjogren’s antibodies A and B, normal lipase (4 U/L; reference range: 7-60 U/L), normal C-telopeptide (534 pg/mL; reference range: 25-573 pg/mL for premenopausal) and a complete blood count with peripheral blood smear revealing microcytic hypochromic anemia, mild lymphopenia, and mild monocytosis with adequate reticulocytosis. Serum-free light chains showed a high free kappa (3.58 mg/dL; reference range: 0.33-1.94 mg/dL), normal free lambda (2.59 mg/dL; reference range: 0.57 to 2.63 mg/dL), and a normal free kappa/lambda ratio (1.38; reference range: 0.26-1.65). Ultrasound of the abdomen showed a 7mm non-obstructing calculus in the mid-right kidney. Chest X-ray revealed no hilar lymphadenopathy or acute process (Figure 1).

Considering the patient’s history, physical exam findings, and diagnostic work up until then, further investigations to rule out malignant lymphoma and breast cancer were carried out.

**Figure 2:**
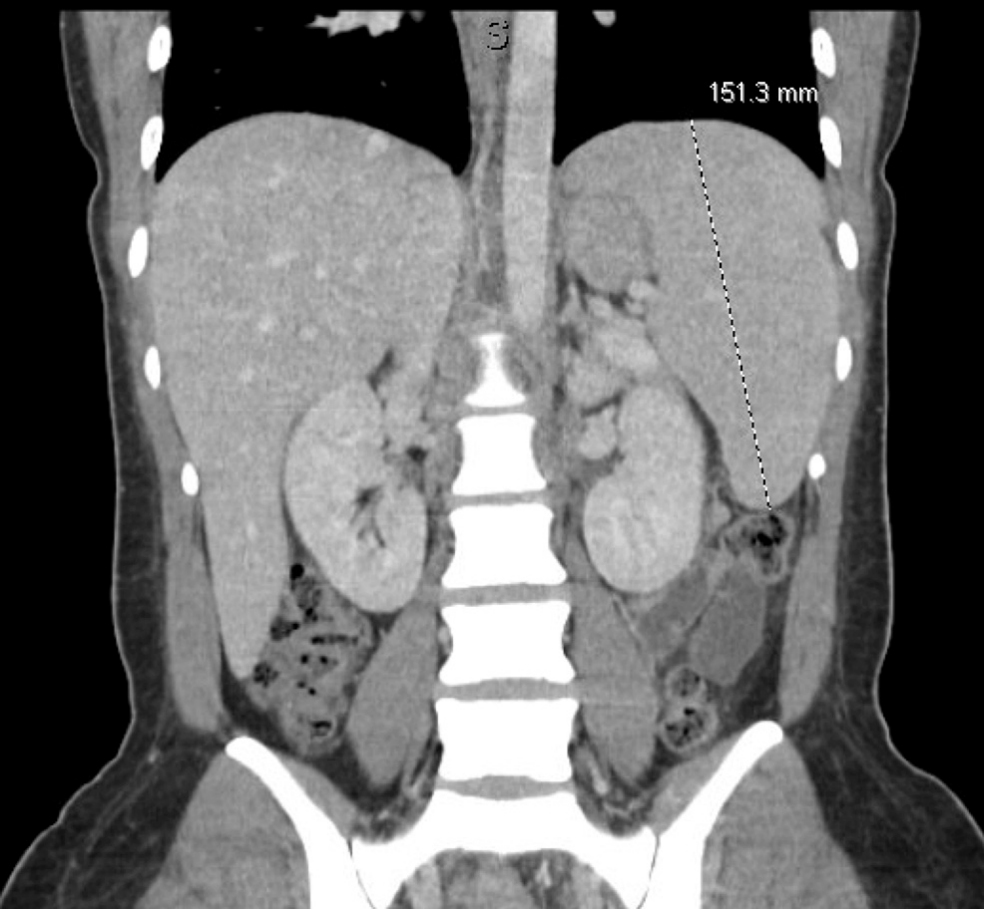
CT of the abdomen demonstrating splenomegaly.

**Figure 3:**
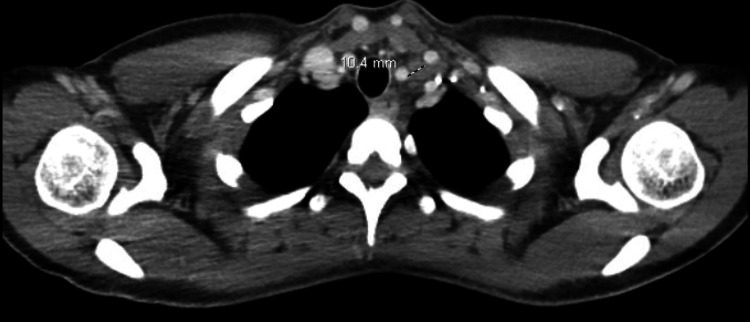
CT of the chest showing a mildly enlarged superior mediastinal lymph node between the left subclavian and left common carotid artery.

**Figure 4:**
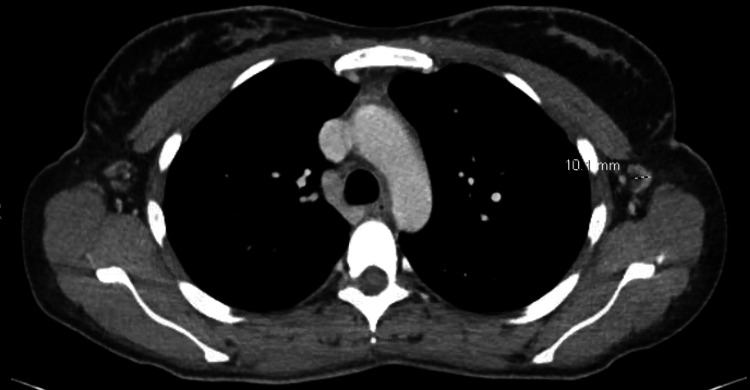
CT of the chest demonstrating left axillary lymphadenopathy.

Serum tumor markers CA 15-3 (28.8 U/mL; reference range: 0.0-31.9 U/mL), CA 27.29 (38.4 U/mL; reference range: <38.6 U/mL), and CEA (0.7 ng/mL; reference range: 0.0-3.0 ng/mL) were all within normal range. A CT of the chest, abdomen, and pelvis with contrast revealed mild nonspecific splenomegaly with nonspecific upper mediastinal, left hilar, and left supraclavicular adenopathy up to 10 mm and mildly enlarged upper abdominal and retroperitoneal lymph nodes up to 10-17 mm (Figures 2-4).

**Figure 5:**
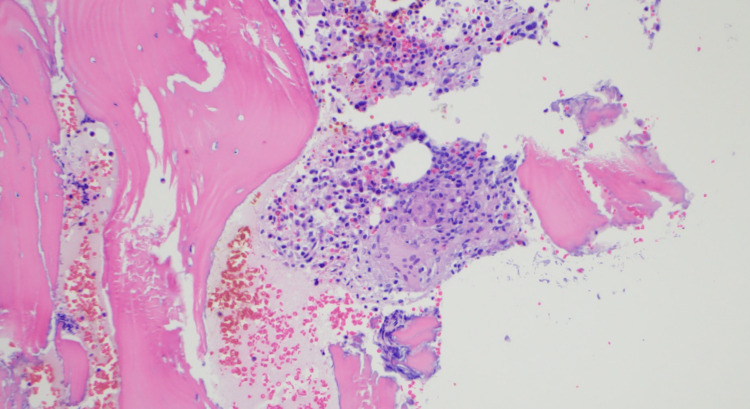
Bone marrow biopsy demonstrating small non-caseating granulomas.

A bone scan showed no suspicious radiotracer uptake within the axial or appendicular skeleton. PET scan showed moderate uptake involving the bilateral parotid and submandibular glands, redemonstration of the patient’s splenomegaly, as well as left supraclavicular, thoracic, and upper abdominal lymphadenopathy with mild FDG uptake. Because of the absence of palpable lymph nodes on physical exam and no probable targets for excisional biopsy seen on imaging, a bone marrow biopsy was performed and showed a normocellular bone marrow with trilineage hematopoiesis and small noncaseating granulomas (Figure 5). No evidence of malignancy was noted.

**Figure 6:**
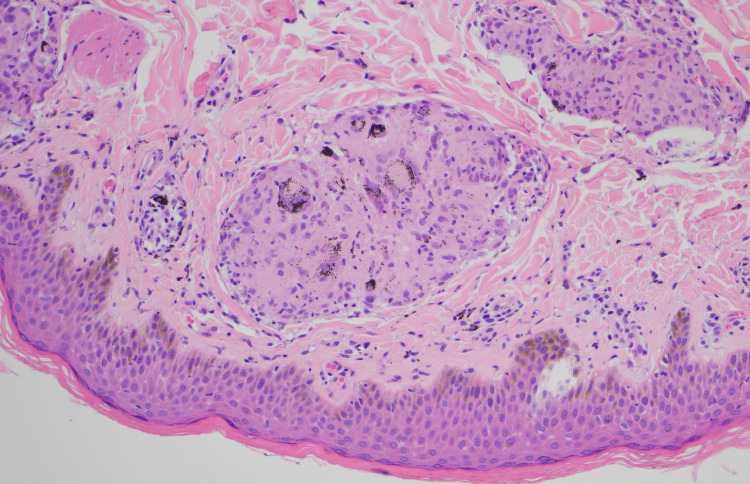
Skin biopsy demonstrating non-caseating granulomas.

An infectious workup showed a past EBV infection (EBV VCA IgG >750 U/mL (reference range: 0.0-17.9 U/mL), EBV nuclear antigen IgG 443.0 U/mL (reference range: 0.0-17.9 U/mL); otherwise, the workup was negative for TB, syphilis, coccidioides, histoplasma, and blastomyces. At this point, the patient reported that she had noticed skin changes in the tattoo on her right upper arm in the last few months. She reported that it became more pruritic and mildly painful, with associated skin thickening and scaling. A biopsy of the tattoo revealed multiple well-formed, non-caseating granulomas containing tattoo pigment in the dermis. It was negative for fungal elements or bacterial colonies (Figure 6). Based on the patient’s history, physical examination, and laboratory findings, a diagnosis of extrapulmonary sarcoidosis with cutaneous, splenic, lymph node, and bone marrow involvement was made.

Our patient was started on oral prednisone 40 mg daily, after which she was started on a long-term tapering regimen that was completed after seven months. Shortly after starting treatment, she clinically improved. Of note, a month after starting steroid therapy, she was started on hydroxychloroquine 200 mg daily as a steroid-sparing agent as she started experiencing increased appetite and weight. As of the time of this writing, the patient has had no recurrence of her symptoms. A repeat CT of her chest six months after starting treatment also demonstrated an interval decrease in lymphadenopathy and splenomegaly.

## Discussion

Sarcoidosis is a systemic granulomatous disease of unknown etiology that affects people all over the world. Most patients will experience a benign clinical course, but there are factors such as age, sex, and ethnicity that play a role in a patient’s overall prognosis. In the United States specifically, black women have been found to have a higher incidence and prevalence of sarcoidosis [11] and a higher mortality rate [12]. Because of its variable presentation and multisystemic involvement, it is largely a diagnosis of exclusion. It relies on a compatible clinical presentation, findings of non-necrotizing granulomatous inflammation in one or more tissue samples, and the exclusion of alternative causes of granulomatous disease [13]. Hypercalcemia affects anywhere between 10% and 20% of patients with sarcoidosis. It results due to an excess conversion of 25(OH) vitamin D to 1,25(OH) vitamin D by macrophage 1-a-hydroxylase within granulomas. Hypercalcemia itself has been shown to be associated with more severe disease phenotypes, and if hypercalciuria occurs, it predisposes to nephrolithiasis as well [6,8]. In light of this, it is imperative to remember that this mechanism of hypercalcemia is not specific to sarcoidosis, and so it is imperative that other etiologies be first considered.

In our patient, the findings of non-PTH-dependent hypercalcemia, increased levels of serum IL-2 and ACE, and lymphadenopathy on imaging made malignant lymphoma the most important differential diagnosis to be ruled out. Fortunately, the biopsy did not reveal any findings of malignancy. Other etiologies of granulomatous inflammation in the bone marrow that must be considered include infectious causes like tuberculosis and fungal organisms, autoimmune diseases like IgG4-related disease and Langerhans cell histiocytosis, and drug-induced granulomatous disease [13]. Our patient’s history and diagnostic work were not compatible with all these other etiologies. She did have splenomegaly on imaging and evidence of past EBV infection. However, splenomegaly in EBV infection usually occurs in the acute setting and resolves within four to six weeks [14]. Splenic involvement in sarcoidosis, on the other hand, is often clinically silent and shows no laboratory abnormalities [5]. Given this and her overall clinical presentation, we assessed that sarcoidosis is the most logical diagnosis.

The lungs are the most common organ affected in sarcoidosis, occurring around 90% of the time [15]. Extrapulmonary involvement can occur, and organ involvement has been found to vary depending on factors such as age, gender, and race [15,16]. However, only about 2% to 8% of patients have extrapulmonary sarcoidosis without pulmonary involvement [15,17]. We present a rare case of extrapulmonary sarcoidosis without pulmonary involvement, involving the bone marrow, skin, lymph nodes, and spleen.

Furthermore, bone marrow involvement itself is rare, and differing patterns of bone marrow involvement were observed according to ethnicity. In the ACCESS study [15], where 44% of the 736 patients enrolled were African Americans, bone marrow involvement was present in only 3.9% of patients, while studies conducted in Japan, China, and India did not report it [16]. In a cohort study of 640 patients diagnosed with sarcoidosis over 40 years in Barcelona, Spain, where the majority of the patients were Caucasian, there was no BM involvement at the time of diagnosis and only 0.3% were diagnosed with it in the follow-up period, which spanned years [17]. In a study examining 1686 patients with biopsy-proven sarcoidosis, James et al. [18] found that BM involvement occurred more frequently in those with nonpulmonary sarcoidosis (12.1%) compared to those with pulmonary sarcoidosis (6%). Despite sarcoidosis being one of the more common causes of BM granulomas, it is rare for sarcoidosis with BM involvement to have granulomas [19]. For example, in a single-center study that examined BM biopsies from sarcoidosis patients between 1994 and 2002, only 10% revealed granulomas [19]. These patients can present with hematologic abnormalities secondary to BM infiltration, but they may also have no symptoms and have normal hematologic parameters as well.

In contrast to BM involvement, the skin is one of the most affected organs outside of the lungs. It can appear as macules, papules, or plaques, and it can mimic many other dermatologic conditions. Given this, cutaneous sarcoidosis can be easily missed or misinterpreted, so the index of suspicion must remain high [20]. This is important because while skin involvement can present as an isolated dermatologic condition, it frequently heralds the diagnosis of multiorgan involvement. Therefore, systemic evaluation is recommended when patients have cutaneous sarcoidosis [4,18,21]. In the case of our patient, her only manifestation was a change in her shoulder tattoo. There is a known association between sarcoidosis and scar tissues, traumatized sites, and tattoos. The exact mechanism remains to be discovered, but proposed mechanisms include koebnerization or, specifically in tattoos, chronic antigenic stimulation that is caused by the pigments in genetically predisposed individuals [20,22].

Given the complexity and multisystemic nature of sarcoidosis, there is no ideal biomarker that would confirm the diagnosis of this disease. Serum biomarkers such as ACE, interleukin-2, chitotriosidase, lyzozymes, and many others have been studied. Serum ACE, which is produced in the epithelioid of the sarcoid granuloma, is perhaps the most well-known. Up to 80% of patients with sarcoidosis will have elevated levels; however, their specificity and sensitivity are poor. Interleukin-2 is a more recent biomarker. It is a cytokine that plays a role in T-cell proliferation. Its utility as a biomarker is still unknown, but it has been suggested that it can be used to identify extrapulmonary involvement in sarcoidosis [23]. In our patient, both serum ACE and interleukin-2 levels were elevated, and we interpreted them in conjunction with all our additional findings.

## Conclusions

Extrapulmonary sarcoidosis is a challenging disease because of its variable presentation and multisystem involvement. While most patients experience a benign clinical course, some patients can experience debilitating consequences when the diagnosis is delayed. This case demonstrates the value of a detailed history and physical exam and highlights the need for tissue diagnosis in an appropriate clinical setting in making the diagnosis of this rare medical condition.
